# Consanguinity decreases risk of breast cancer – cervical cancer unaffected

**DOI:** 10.1054/bjoc.2001.2131

**Published:** 2001-11

**Authors:** S Denic, A Bener

**Affiliations:** Department of Internal Medicine, Department of Community Medicine, Faculty of Medicine and Health Sciences, UAE University, PO Box 17666, AI Ain, United Arab Emirates

**Keywords:** breast cancer, cervical cancer, consanguinity, *BRCA1*, *BRCA2*

## Abstract

Marriages between third-degree and more distant relatives are common in many parts of the world. Offspring of consanguineous parents have increased morbidity and mortality related to recessive gene disorders. In a population with a high frequency of consanguinity, we examined the frequency of breast cancer (related in part to tumour genes) and cervical cancers (related to virus infection) among offspring of consanguineous and non-consanguineous parents. Study was done prospectively in the United Arab Emirates. Selected were married female citizens, ages 40–65, who attended 12 primary health care clinics for whatever reason. In a face-to-face interview, subjects were asked: (a) about consanguineous marriages in family; (b) if they have or have had breast or cervical cancer; (c) about family history of cancer, cancer screening and other parameters. Tumour diagnosis was confirmed by review of medical records. Of 1750 women invited into study, 1445 (79%) could be used in analysis. Among 579 (40%) women of consanguineous and 866 (60%) of non-consanguineous parents there were 24 and 54 with breast cancer, respectively (RR = 0.66, CI 0.42 – 1.06). In the 40 to 50 age group, breast cancer reported 13 of 446 women of consanguineous and 37 of 633 of non-consanguineous parents (RR = 0.50, Cl 0.27 – 0.93). Cervical cancer had 15 women in consanguineous and 32 in non-consanguineous group (RR = 0.70, Cl 0.38 – 1.28). Number of families with history of breast cancer in consanguineous and non-consanguineous group was 21 and 23, respectively (*P* = 0.29). The cancer screening rates and other variable values had fairly balanced distribution between the 2 groups. Having consanguineous parents decreases the risk of breast cancer especially in younger women, risk of cervical cancer being unaffected. © 2001 Cancer Research Campaign http://www.bjcancer.com


*British Journal of Cancer* (2001) **85**(11), 1675–1679
© 2001 Cancer Research Campaign
doi: 10.1054/ bjoc.2001.2131, available online at http://www.idealibrary.com on IDEAL

### Consanguinity decreases risk of breast cancer – cervical cancer unaffected

**S Denic[1] and A Bener[2]**

[1]Department of Internal Medicine; [2]Department of Community Medicine, Faculty of Medicine and Health Sciences, UAE University, PO Box 17666, Al Ain, United Arab Emirates

**Summary** Marriages between third-degree and more distant relatives are common in many parts of the world. Offspring of consanguineous parents have increased morbidity and mortality related to recessive gene disorders. In a population with a high frequency of consanguinity, we examined the frequency of breast cancer (related in part to tumour genes) and cervical cancers (related to virus infection) among offspring of consanguineous and non-consanguineous parents. Study was done prospectively in the United Arab Emirates. Selected were married female citizens, ages 40–65, who attended 12 primary health care clinics for whatever reason. In a face-to-face interview, subjects were asked: (a) about consanguineous marriages in family; (b) if they have or have had breast or cervical cancer; (c) about family history of cancer, cancer screening and other parameters. Tumour diagnosis was confirmed by review of medical records. Of 1750 women invited into study, 1445 (79%) could be used in analysis. Among 579 (40%) women of consanguineous and 866 (60%) of non-consanguineous parents there were 24 and 54 with breast cancer, respectively (RR = 0.66, CI 0.42 – 1.06). In the 40 to 50 age group, breast cancer reported 13 of 446 women of consanguineous and 37 of 633 of non-consanguineous parents (RR = 0.50, CI 0.27 – 0.93). Cervical cancer had 15 women in consanguineous and 32 in non-consanguineous group (RR = 0.70, CI 0.38 – 1.28). Number of families with history of breast cancer in consanguineous and non-consanguineous group was 21 and 23, respectively ($P$ = 0.29). The cancer screening rates and other variable values had fairly balanced distribution between the 2 groups. Having consanguineous parents decreases the risk of breast cancer especially in younger women, risk of cervical cancer being unaffected. © 2001 Cancer Research Campaign http://www.bjcancer.com

**Keywords**: breast cancer; cervical cancer; consanguinity; *BRCA1*; *BRCA2*

In the Arab world, man marries his paternal uncle's daughter so often that another name for a wife is 'my uncle's daughter' even if couples are not related. Marrying a relative is practiced in many parts of the world and only recently the rate has declined in Western countries to below 1% of all marriages (Khlat, 1977 Harper, 1993; Jaber et al, 1998). Consanguineous marriages are between a third-degree and more distant relatives. In North Africa and the Middle East, weddings between first cousins (the children of two brothers or sisters or brother and sister) represent 50% to 86% of all consanguineous weddings (Khlat, 1977). In the Arabian Gulf countries every other marriage is consanguineous and in the United Arab Emirates (UAE) the frequency has increased in the last generation (Al Gazali et al, 1997). In other Arab countries, Sudan, Egypt, Israel and Turkey between 21% and 51% of all marriages are consanguineous (Basaran et al, 1989; Saha et al, 1990; Abdulrazzaq et al, 1997; Al Gazali et al, 1997). Marriages between biological relatives are also common in Iran, Pakistan, India, Brazil and Japan (Imaizumi, 1986; Sureender et al, 1998). A few hundreds of millions of adults and their children worldwide are in consanguineous families.

The consanguineous couples have an increased frequency of abortions, stillbirths, postnatal mortality and children with congenital malformation, mental retardation, hearing defects and autosomal recessive disorders. While cancer is generally not associated with consanguinity (Khlat, 1997; Jaber et al, 1998), we have recently found that consanguinity alters the risk of lymphoid malignancies (Bener et al, 2001).

The tumour-suppressor and mutator genes are recessive and the presence of 2 identical alleles in an individual is required to accomplish a step in a multi-step carcinogenesis. In a model of cancer development, zygote receives one cancer gene from one of the germ-cells and another is acquired later in life as a somatic mutation. For example, when *BRCA1* and *BRCA2* genes are inherited, the risk of breast cancer is increased several-fold, leading to development of cancer at an earlier age. These patients also have a strong family history of cancer (Anonymous, 1999). Nonetheless, if a recessive tumour gene is present in a family and 2 members of such a family conceive an offspring, theoretically, a child could be born with 2 recessive tumour genes, i.e. a congenital step of carcinogenesis. Such an individual would be expected to develop cancer earlier in life. However, if a gene causing lethal illness develops before an individual could reproduce, such a deleterious gene would be lost from a population (Khlat, 1997). Thus a long-term practice of consanguinity may decrease the frequency of recessive tumour genes, theoretically, leading to a lower incidence of cancer in a consanguineous population. In this study we examine the possible effect of inbreeding on the risk of breast and cervical cancers in a population with a high rate of consanguinity.

*Received 13 March 2001*
*Revised 3 September 2001*
*Accepted 11 September 2001*

*Correspondence to:* A Bener

### MATERIALS AND METHODS

This study is a cross-sectional community-based survey conducted between August 1999 and February 2000 in the city of Al Ain, United Arab Emirates.

### Study subjects

Study subjects were consecutive female subjects who attended 12 primary health care clinics for any reason. Selected were married women who are UAE nationals, ages 40 to 65. Targeted sample size was 1750 individuals or one third of estimated population size of the given sex and age. The sampling from the clinics was proportional to 25–75% of urban to semi-urban distribution of population.

### Questionnaire and interview

The questionnaire was made in Arabic with single forward and back translations to English being made to ensure its linguistic validity. The subjects were asked if they, their parents and their husbands' parents (in-laws) are consanguineous and if they have or had breast or cervical cancer. They were asked about breast and cervical cancer among their mothers, sisters and daughters. Family history was considered positive for breast cancer if at least one first-degree female relative had a breast carcinoma. Medical records were reviewed to confirm diagnosis of breast and cervical cancer in study subjects with history of malignancy. Socio-demographic information (age, education, occupation, number of children, annual number of visits to clinic, family income) was collected as well as data on breast and cervical cancer screening performed. Age of subjects is at the time of interview. Questionnaires were administered during the face-to-face interviews, which were conducted in Arabic by a health educator and one of 10 qualified nurses. In a sample of 50 subjects, the validity and reliability of a questionnaire was tested by comparing the number of reported cancer screening tests with the number of documented tests in medical records.

### Analysis

The data were coded and entered into a computer using the Statistical Packages for Social Sciences (Norusis, 1996). Data are expressed as mean and standard deviation (SD) unless otherwise stated. The Student's *t*-test was used to ascertain the significant differences between mean values of 2 continuous variables and Mann–Whitney for non-parametric distribution. $\chi^2$ was performed to ascertain the association between 2 or more categorical variables. In 2 × 2 tables, the Fisher exact test (2-tailed) was used instead of $\chi^2$, in particular, when sample size was small. The relative risk (RR) and their 95% confidence interval (CI) were obtained by using Mantel–Haenszel test. Multiple logistic regression analysis was used to assess the relationship between breast cancer as the dependent variable and other socio-demographic and biological factor as independent variables. Those variables found to be significant were used in multiple logistic model. Logistic regression results are reported as RR and CI (derived from likelihood ratios and its standard error) along with *P* values (derived from likelihood ratios statistics which have a $\chi^2$ distribution). The level $P < 0.05$ was considered as the cut-off value for significance. κ coefficients were used to examine agreement between medical records and self report, and values above 0.75 are taken to indicate excellent agreement, between 0.4 and 0.75 good agreement and below 0.4 poor agreement.

### RESULTS

Of 1750 women invited to participate in the study, 21% (305) refused or gave incomplete answers to questions and were removed from analysis. The self-reported cancer screenings were in high agreement with medical records data (κ = 0.82). All subjects who reported the breast and cervical cancer had the same confirmed in the medical records.

### Consanguinity rates

Of 1445 analysed subjects, 40% (579) had consanguineous and 60% (866) had non-consanguineous parents. Among the subjects' in-laws, 41% (594) were consanguineous. The subjects' parents and in-laws belong to the same generation and their consanguinity rates were not different ($P = 0.57$). The number of study subjects who themselves married a relative was 747 (52%), a significantly higher rate than in their parents ($P < 0.0001$). Parental consanguinity did not increase the chance of female offspring entering consanguineous marriage but did increase the chance of male offspring (husband) entering consanguineous marriage (Table 1). 200 subjects (14%) reported that they themselves, their parents and in-laws are all in consanguineous matrimony.

### Breast and cervical cancer risks

Breast cancer frequencies among women whose parents were consanguineous and non-consanguineous are shown in Table 2. Overall, there is a borderline protective effect of consanguineous parents on risk of breast cancer, and this is significant among those aged 40–50. The mean age of 78 women with history of breast cancer whose parents were consanguineous versus non-consanguineous was 48.6 vs. 46.9 years ($P = 0.31$); in the age group of 40 to 50 the mean age was 42.9 vs. 43.8 years ($P = 0.25$), and in the age group of 51 to 65 the mean age was 55.4 vs. 53.7 years ($P = 0.29$). Cervical cancer rate was not different between women of consanguineous and non-consanguineous parents (Table 2).

### Family history of cancer and sociodemographics

Family history of breast cancer overall was not different between consanguineous and non-consanguineous groups (Table 2). In the 40 to 50 age group, 15 of 446 consanguineous and 18 of 633 non-consanguineous families had positive history of breast cancer ($P = 0.63$). In the 51 to 65 age group, 6 of 133 consanguineous and

**Table 1** Consanguinity rates in 2 generations reported by 1445 married women

| | Wife Parents | | | Husband parents | | |
|---|---|---|---|---|---|---|
| **Wife marriages** | **Consanguin.** | **Non-consanguin.** | **RR (95% CI)** | **Consanguin.** | **Non-consanguin.** | **RR (95% CI)** |
| Overall | 579 (40%) | 866 (60%) | | 594 (41%) | 851 (59%) | |
| Consanguineous | 301 | 446 | 1.01 (0.91–1.12) | 358 | 389 | 1.32 (1.20–1.45) |
| Non-consanguineous | 278 | 420 | | 236 | 462 | |

 *© 2001 Cancer Research Campaign*

**Table 2** Comparative characteristics of women of consanguineous and non-consanguineous parents

| | | Parents | | | |
|---|---|---|---|---|---|
| | | **Consanguineous** *n* = 579 | **Non-consanguineous** *n* = 866 | **RR (95% CI)** | ***P*** |
| Breast cancer | | | | | |
| Overall | | 24 (4.1%) | 54 (6.2%) | 0.66 (0.42 – 1.06) | 0.08 |
| Age 40 – 50 | Cancer | 13 (2.9%) | 37 (5.8%) | 0.50 (0.27 – 0.93) | 0.02 |
| | No Cancer | 433 | 596 | | |
| Age 51 – 65 | Cancer | 11 (8.2%) | 17 (7.3%) | 1.13 (0.55 –2.35) | 0.89 |
| | No Cancer | 122 | 216 | | |
| Cervical cancer | | 15 (2.6%) | 32 (3.7%) | 0.70 (0.38 – 1.28) | 0.25 |
| Education (years) | | | | | |
| 0 | | 342 (59.1%) | 486 (56.1%) | 1.00 | |
| 1 – 6 | | 161 (27.8%) | 230 (26.6%) | 1.00 (0.86 – 1.15) | 0.96 |
| 6 | | 76 (13.1%) | 150 (17.3%) | 0.81 (0.67 – 0.99) | 0.04 |
| Occupation | | | | | |
| Employed | | 67 (11.6%) | 109 (12.6%) | 0.92 (0.77 – 1.15) | 0.56 |
| House wife | | 512 (88.4%) | 757 (87.4%) | 1.00 | |
| Net monthly income (US $) | | | | | |
| < 1359 | | 177 (30.6%) | 255 (29.4%) | 1.00 | |
| 1359 – 2717 | | 149 (25.7%) | 223 (25.8%) | 0.98 (0.83 – 1.16) | 0.79 |
| > 2717 | | 253 (43.7%) | 388 (44.8%) | 0.96 (0.83 – 1.12) | 0.62 |
| Had mammogram* | Yes | 64 (11.5%) | 77 (9.5%) | 1.22 (0.89 – 1.66) | 0.22 |
| | No | 491 | 735 | 1.00 | |
| Had pap test* | Yes | 97 (17.2%) | 118 (14.1%) | 1.22 (0.95 – 1.56) | 0.12 |
| | No | 467 | 716 | 1.00 | |
| Family history of breast ca. | | | | | |
| | Positive | 21 (3.6%) | 23 (2.6%) | | 0.29 |
| | Negative | 558 | 843 | | |
| Mean age (±SD) | | | | | |
| Overall | | 46.1 (6.0) | 46.8 (6.0) | | 0.05 |
| Age 40 – 50 | | 43.4 (2.9) | 43.7 (2.8) | | 0.38 |
| Age 51 – 65 | | 55.2 (4.8) | 55.0 (4.6) | | 0.73 |
| Mean no. clinic visits (±SD) | | | | | |
| Overall | | 9.3 (6.8) | 10.2 (7.9) | | 0.04 |
| Age 40 – 50 | | 9.0 (6.5) | 9.7 (7.6) | | 0.14 |
| Age 51 – 65 | | 10.2 (7.7) | 11.4 (8.8) | | 0.20 |
| Mean no. of children (±SD) | | 7.1 (3.2) | 7.1 (3.1) | | 0.73 |

*Patients with cancer detectable by the screening test were excluded from analysis as all had the test.

**Table 3** Risk factors for breast cancer using multivariate logistic regression analysis

| | **RR** | **(95% CI)** | ***P*** |
|---|---|---|---|
| Overall | | | |
| Parental consanguinity | 0.66 | (0.40 – 1.08) | 0.097 |
| Education | 0.83 | (0.71 – 0.98) | 0.024 |
| Age | 0.96 | (0.93 – 1.00) | 0.047 |
| Number of clinic visits | 1.07 | (1.02 – 1.12) | 0.005 |
| Age 40 – 50 | | | |
| Parental consanguinity | 0.49 | (0.26 – 0.94) | 0.032 |
| Education | 0.76 | (0.62 – 0.92) | 0.005 |
| Age | 1.0 | (0.91 – 1.11) | 0.970 |
| Number of clinic visits | 1.03 | (0.98 – 1.08) | 0.308 |
| Age 51 – 65 | | | |
| Parental consanguinity | 1.08 | (0.48 – 2.43) | 0.850 |
| Education | 1.0 | (0.73 – 1.36) | 0.984 |
| Age | 1.03 | (0.49 – 1.13) | 0.531 |
| Number of clinic visits | 1.16 | (1.06 – 1.27) | 0.002 |

5 of 233 non-consanguineous families had positive history of breast cancer ($P = 0.20$). Overall, positive family history of breast cancer had 4 (5%) of 78 women with breast cancer and 40 (3%) of 1367 women without breast cancer, a non-significant difference ($P = 0.27$).

The socio-demographic characteristics of the offspring of consanguineous and non-consanguineous parents are shown in Table 2. Overall, the education, age and number of clinic visits were lower among women of consanguineous than of non-consanguineous parents. The results of multivariate analysis are given in Table 3.

### DISCUSSION

The risk of breast cancer among younger Arabian women whose parents are consanguineous is significantly lower than the risk of women whose parents are non-consanguineous. For the whole group, the risk of breast cancer was reduced by parental consanguinity but reduction did not reach statistical significance. Parental consanguinity had no effect on the frequency of cervical carcinoma that is related to viral infection and not to inheritance of genes.

The theoretical framework that explains decreased prevalence of breast cancer among offspring of consanguineous parents must explain the apparent loss of 'breast cancer patients' from the consanguineous subgroup of a population. The loss could be

© 2001 Cancer Research Campaign 

accounted for by the increased rates of abortion, stillbirth, perinatal and child mortality found in consanguineous families (Asha Bai et al, 1981; Basaran et al, 1989; Shami et al, 1989; Saha et al, 1990; Powell et al, 1995; Grant and Bittles, 1997; Shah, 1997; Hussain, 1998; Jaber et al, 1998; Stoltenberg et al, 1999). We did not examine the rate of abortions, stillbirths or early child death in our study, but previous study made of in the same population as ours showed an increased overall fetal wastage in consanguineous families (Abdulrazzaq et al, 1997). The number of children in our study was the same for consanguineous and non-consanguineous families. These numbers may not reflect the loss of 'future breast cancer patients' because fertility of consanguineous couples may be 'compensatively' increased (Hussain, 1998). Another link between increased fetal wastage and mortality on one side, and breast cancer on the other is discovery that the same genes are active in both embryogenesis and oncogenesis. For example, *BRCA1* gene was found to be active in different tissues during human embryogenesis (Pavelic et al, 1991). The *BRCA1* and *BRCA2* knock-out mice are not viable and are aborted during embryonic life (Hakem et al, 1998), suggesting that the same may cause abortion or stillbirths in humans. Further, *HER-2* gene is expressed in a third of breast cancers and is believed to be important for embryogenesis (Alroy and Yarden, 1997). Zygotic or congenital homozygosity for some recessive oncofetal genes may impair embryogenesis leading to abortion or contribute to stillbirth or premature child death, eliminating individuals with the same 2 oncofetal genes (natural knock-outs). This suggests that *BRCA1/2*, which cause more breast cancers in younger than older women, are infrequent in populations with a long history and high rate of consanguinity.

If an offspring of consanguineous parents homozygous for a recessive tumour gene dies before it biologically reproduces, the frequency of such a gene would decrease. The decrease of gene frequency would be more significant in a population with a high rate and long history of consanguinity. As a result, a decreased incidence of a tumour in such a population would be expected. Indeed, age-standardized incidence of breast cancer for 1998 in our native population was 15.5 per 100 000 (unpublished data of UAE Cancer Registry). The populations of Kuwait and Saudi Arabia have the same high consanguinity rate and their 1998 breast cancer age-standardized incidence was 31.8 and 18.6 per 100 000, respectively (Anonymous, 2000). In North America and Western Europe where the consanguinity rate is less then 1%, breast cancer incidence is 86 and 68 per 100 000, respectively (Parkin et al, 1999). Thus Gulf countries with consanguinity rate of over 50% when compared with developed countries with consanguinity rate of less than 1%, have several times lower incidence of breast cancer. Japan with a long past history of consanguinity and current 4% prevalence of consanguineous marriages and India with a high but geographically varying consanguinity rate both have an incidence rate below 30 per 100 000 (Parkin et al, 1999). Although reproductive, dietary, demographic and other risk factors account for some differences between the countries, current and past history of consanguinity, particularly its long-term practice, may need to be considered in explanation of observed variability.

Our data suggest that the rate of consanguineous marriages in UAE has increased over one generation by 25%. The same was found in another study (Al Gazali et al, 1997) and could be related to the enormous increase in the country's wealth over the last couple of decades brought by exploitation of oil and a desire of families to preserve it. While women whose parents were consanguineous were not more likely to enter into consanguineous marriage themselves, their husbands were more likely to marry a relative if their parents were consanguineous (RR = 1.32). This finding is explainable by social dynamics in this strongly patriarchal society in which males' parents choose the bride. It also means that inbreeding is stronger patrilineally.

Family history of breast cancer is a known risk factor that may, if unbalanced between comparison groups, skew the results. We found that family history of breast was not significantly different between groups with consanguineous and non-consanguineous parents. Further, there was no expected difference in the frequency of positive family history of breast cancer between women with and without breast carcinoma (5% and 3%, respectively). This could result from insufficient power of study to detect a difference. However, it is possible that high rate and long history of consanguinity have changed genomics of breast cancer in this population by eliminating one and making relatively more common other cancer genes. This suggests that *BRCA1* and *BRCA2* genes may be less frequent in highly consanguineous populations and is supported by the finding of mitigated family history in breast cancer probands.

The result could be a chance finding or an outcome of bias caused by 21% drop out of study subjects. The recall bias is unlikely in the study as both groups were questioned in identical manner. Chances that other variables may have affected the result are low as our study population is homogeneous in that all subjects are nationals of narrow age range and Muslims. They belong to a culture in which almost none consume alcohol and a negligible number smokes, further contributing to homogeneity of the study population. However, other risk factors like age at menarche and oestrogen intake were not considered although they may affect the result. Fewer clinic visits among offspring of consanguineous parents could be a chance phenomenon caused by multiple comparisons; it was not a significant variable in the 40 – 50 age group in which parental consanguinity was found to significantly reduce risk of breast cancer. Different socio-demographic parameters and breast and cervical cancer screening were not significantly different between consanguineous and non-consanguineous groups, making influence of environmental factors and detection bias less likely (Table 2). The finding of lower mean age and education level among offspring of consanguineous parents cannot be excluded as a bias but others have noted association of lower age and education, and consanguinity (Sureender et al, 1998). A fewer breast cancer cases in consanguineous than in non-consanguineous group may result if breast cancer patients in former group have a shorter survival than those in later. However, there is no evidence to suggest true existence of such a biological difference. We acknowledge that relatively low power of the study resulted in wide confidence intervals.

In conclusion, this study suggests that parental consanguinity decreases the risk of breast cancer in younger women. The *BRCA1/2* are risk factors of breast cancer especially in younger women and consanguinity alter tumour genomics. Some of the genes are important in development of both breast cancer and normal embryo. An oncofetal gene homozygosity resulting from consanguinity impairs embryogenesis, perinatal or postnatal development, and leads to increased fetal wastage and child mortality in consanguineous couples. Some of these lost individuals are actually 'lost breast cancer patients'. The finding that

 *© 2001 Cancer Research Campaign*

parental consanguinity reduces the risk of breast cancer should be confirmed, as it may be important for consanguineous families worldwide.

### ACKNOWLEDGEMENT

The authors thank Dr Hussain Saadi for critical review of the manuscript and acknowledge contribution of the reviewers in preparation of the manuscript. This project was funded by the grant (NP/99/5) to one of us (AB) from the Faculty of Medicine and Health Sciences, UAE University, Al Ain, Abu Dhabi, UAE.

### REFERENCES

Abdulrazzaq YM, Bener A, Al-Gazali LI, Al-Khayat AI, Micallef R and Gaber T (1997) A study of possible deleterious effects of consanguinity. *Clin Genet* **51**: 167–173

Al Gazali LI, Bener A, Abdulrazzaq YM, Micallef R, Al-Khayat AI and Gaber T (1997) Consanguineous marriages in the United Arab Emirates. *J Biosoc Sci* **29**: 491–497

Alroy I and Yarden Y (1997) The ErbB signaling network in embryogenesis and oncogenesis: signal diversification through combinatorial ligand-receptor interactions. *FEBS Lett* **410**: 83–86

Anonymous (1999) Molecular biology. In *Oncology: Medical knowledge self-assessment program*, pp 20–51. American College of Physicians–American Society of Internal Medicine: Philadelphia

Anonymous (2000) Cancer incidence report Gulf Cooperation Council (GCC) Countries 1998. Executive Office for GCC Ministries (draft)

Asha Bai PV, John TJ and Subramaniam VR (1981) Reproductive wastage and developmental disorders in relation to consanguinity in south India. *Trop Geogr Med* **29**: 275–280

Basaran N, Hassa H, Basaran A, Artan S, Stevenson JD and Sayli BS (1989) The effect of consanguinity on the reproductive wastage in the Turkish population. *Clin Genet* **36**: 168–173

Bener A, Denic S and Al-Mazrouei M (2001) Consanguinity and family history of cancer in children with leukemia and lymphomas. *Cancer* **92**: 1–6

Grant JC and Bittles AH (1997) The comparative role of consanguinity in infant and childhood mortality in Pakistan. *Ann Hum Genet* **61**: 143–149

Hakem R, de la Pompa JL and Mak TW (1998) Developmental studies of BRCA1 and BRCA2 knock-out mice. *J Mammary Gland Biol Neoplasia* **3**: 431–445

Harper PS (1993) *Practical genetic counseling*. Butterworth-Heinemann Ltd: Oxford

Hussain R (1998) The role of consanguinity and inbreeding as a determinant of spontaneous abortion in Karachi, Pakistan. *Ann Hum Genet* **62**: 147–157

Imaizumi Y (1986) A recent survey of consanguineous marriages in Japan. *Clin Genet* **30**: 230–233

Jaber L, Halpern GJ and Shohat M (1998) The impact of consanguinity worldwide. *Commun Genet* **1**: 12–17

Khlat M (1997) Endogamy in the Arab World. In: *Genetic Disorders Among Arab Populations*, Teebi AS and Farag TI (ed) pp 63–80. Oxford Monographs on medical genetics. No.30. Oxford University Press: New York

Norusis MJ (1996) SPSS Inc. SPSS/PC + for Windows, Base System and Advanced Statistic User's Guide, Window version 10. Chicago, Illinois

Parkin MD, Pisani P and Ferlay J (1999) Incidence of breast cancer in females by world region. *CA: Cancer J Clin* **49**: 33–64

Pavelic K, Slaus NP and Spaventi R (1991) Growth factors and proto-oncogenes in early mouse embryogenesis and tumorogenesis. *Int J Dev Biol* **35**: 209–214

Saha N, Hamad RE and Mohamed S (1990) Inbreeding effects on reproductive outcomes in a Sudanese population. *Hum Hered* **40**: 208–212

Shah GH (1997) Consanguinity and child mortality: the risk faced by families in Pakistan. *http://www.cpc.unc.edu/pubs/paa _ papers/1997shah.html*

Shami SA, Schmitt LH and Bittles AH (1989) Consanguinity related prenatal and postnatal mortality of the populations of seven Pakistani Punjab cities. *J Med Genet* **26**: 267–271

Stoltenberg C, Magnus P, Skrondal A and Lie RT (1999) Consanguinity and recurrence risk of stillbirth and infant death. *Am J Public Health* **89**: 517–523

Sureender S, Prabakaran B and Khan AG (1998) Mate selection and its impact on female marriage age, pregnancy wastages, and first child survival in Tamil Nadu, India. *Soc Biol* **45**: 289–301

*© 2001 Cancer Research Campaign*

## References

[PDF_00400] Abdulrazzaq Y. M., Bener A., al-Gazali L. I., al-Khayat A. I., Micallef R., Gaber T. (1997). A study of possible deleterious effects of consanguinity.. Clin Genet.

[PDF_00406] Alroy I., Yarden Y. (1997). The ErbB signaling network in embryogenesis and oncogenesis: signal diversification through combinatorial ligand-receptor interactions.. FEBS Lett.

[PDF_00414] Asha Bai P. V., John T. J., Subramaniam V. R. (1981). Reproductive wastage and developmental disorders in relation to consanguinity in south India.. Trop Geogr Med.

[PDF_00417] Başaran N., Hassa H., Başaran A., Artan S., Stevenson J. D., Sayli B. S. (1989). The effect of consanguinity on the reproductive wastage in the Turkish population.. Clin Genet.

[PDF_00420] Bener A., Denic S., Al-Mazrouei M. (2001). Consanguinity and family history of cancer in children with leukemia and lymphomas.. Cancer.

[PDF_00422] Grant J. C., Bittles A. H. (1997). The comparative role of consanguinity in infant and childhood mortality in Pakistan.. Ann Hum Genet.

[PDF_00424] Hakem R., de la Pompa J. L., Mak T. W. (1998). Developmental studies of Brca1 and Brca2 knock-out mice.. J Mammary Gland Biol Neoplasia.

[PDF_00432] Imaizumi Y. (1986). A recent survey of consanguineous marriages in Japan.. Clin Genet.

[PDF_00434] Jaber L., Halpern G. J., Shohat M. (1998). The impact of consanguinity worldwide.. Community Genet.

[PDF_00441] Parkin D. M., Pisani P., Ferlay J. (1999). Global cancer statistics.. CA Cancer J Clin.

[PDF_00443] Pavelić K., Pecina Slaus N., Spaventi R. (1991). Growth factors and proto-oncogenes in early mouse embryogenesis and tumorigenesis.. Int J Dev Biol.

[PDF_00445] Saha N., Hamad R. E., Mohamed S. (1990). Inbreeding effects on reproductive outcome in a Sudanese population.. Hum Hered.

[PDF_00449] Shami S. A., Schmitt L. H., Bittles A. H. (1989). Consanguinity related prenatal and postnatal mortality of the populations of seven Pakistani Punjab cities.. J Med Genet.

[PDF_00452] Stoltenberg C., Magnus P., Skrondal A., Lie R. T. (1999). Consanguinity and recurrence risk of stillbirth and infant death.. Am J Public Health.

[PDF_00455] Sureender S., Prabakaran B., Khan A. G. (1998). Mate selection and its impact on female marriage age, pregnancy wastages, and first child survival in Tamil Nadu, India.. Soc Biol.

[PDF_00403] al-Gazali L. I., Bener A., Abdulrazzaq Y. M., Micallef R., al-Khayat A. I., Gaber T. (1997). Consanguineous marriages in the United Arab Emirates.. J Biosoc Sci.

